# Complete Genome Sequence of Klebsiella pneumoniae Myophage May

**DOI:** 10.1128/MRA.00252-19

**Published:** 2019-05-09

**Authors:** Katherine T. Nguyen, Rachele Bonasera, Garret Benson, Adriana C. Hernandez-Morales, Jason J. Gill, Mei Liu

**Affiliations:** a Center for Phage Technology, Texas A&M University, College Station, Texas, USA; Loyola University Chicago

## Abstract

May is a newly isolated myophage that infects multidrug-resistant strains of Klebsiella pneumoniae, a pathogen that is associated with antibiotic-resistant infections in humans. The genome of May has been shown to be similar to that of phage Vi01.

## ANNOUNCEMENT

Klebsiella pneumoniae is linked to respiratory system infections, abscesses, and bacteremia and is a growing global health concern as antibiotic-resistant strains continue to emerge, including the multidrug-resistant strains carrying the *bla*_KPC_ carbapenemase (1). Phages infecting K. pneumoniae may provide treatment options for these infections.

Phage May was isolated from wastewater influent collected in Bryan, Texas, in 2016 using a *bla*_KPC_-containing K. pneumoniae sequence type 258 (ST258) clinical isolate as the host. Host bacteria were cultured on tryptic soy broth or agar (Difco) at 37°C with aeration. Phages were isolated and propagated by the soft agar overlay method (2). Phage genomic DNA was prepared using a modified Promega Wizard DNA cleanup kit protocol as described previously (3). Pooled indexed DNA libraries were prepared using the Illumina TruSeq Nano LT kit, and the sequence was obtained from the Illumina MiSeq platform using the MiSeq V2 500-cycle reagent kit following the manufacturer’s instructions, producing 483,323 paired-end reads for the index containing the phage genome. FastQC 0.11.5 (https://www.bioinformatics.babraham.ac.uk/projects/fastqc/) and FastX Toolkit 0.0.14 (http://hannonlab.cshl.edu/fastx_toolkit/) were used for read quality control, and reads were assembled using SPAdes 3.5.0 (4). The assembled genome was closed by PCR using primers (5′-GGAAATGTCCGGCTGAAGATA-3′ and 5′-GCTGACGCAGTGTGAAATTG-3′) facing off the ends of the assembled contig and Sanger sequencing of the resulting product, with the contig sequence manually corrected to match the resulting Sanger sequencing read. The protein-coding genes were detected with GLIMMER 3.0 and MetaGeneAnnotator 1.0 with manual correction (5, 6), and the tRNAs were identified with ARAGORN 2.36 (7). The gene functions were predicted by similarity detection using BLASTp 2.2.28 (8), and the presence of conserved domains was determined by InterProScan 5.15-5.40 (9). The Center for Phage Technology (CPT) Galaxy (10) and Web Apollo interfaces (11) were used for all analyses using default settings (cpt.tamu.edu).

The genome of phage May was assembled into a closed contig of 159,631 bp with 380.5-fold coverage and a G+C content of 46.8%. The genome contains 214 protein-coding genes with a coding density of 92.7%. The May genome encodes proteins common to many other myophages of the T4 superfamily. A tape measure protein was identified in phage May, but a tape measure chaperone was not detected upstream. An *N*-acetylmuramidase-type endolysin (IPR024408) was identified with a predicted N-terminal peptidoglycan-binding domain (IPR002477); however, no holin or spanin complex could be identified. Using the progressiveMauve algorithm 2.4.0 (12), May was determined to be 79.8% similar to phage 0507-KN2-1 (NC_022343), another phage that infects K. pneumoniae. Phage May also shares 178 proteins with phage Vi01, as determined by a BLASTp search (E < 10^−5^) using the NCBI nonredundant (nr) database, and the arrangement of gene modules within the May genome is similar to that of Vi01 (13). This suggests that phage May is Vi01-like, a member of a group of large myophages that are distantly related to phage T4. A defining feature among Vi01-like phages and consistent in the May genome is the presence of a cluster of predicted tail spike genes (14). The arrangement of four tail spike genes has been predicted to produce umbrella-shaped tail structures (15).

### Data availability.

The genome sequence of phage May was deposited under the GenBank accession no. MG428991. The associated BioProject, SRA, and BioSample accession numbers are PRJNA222858, SRR8788203, and SAMN11259659, respectively.
